# A population genetic analysis of the Critically Endangered Madagascar big-headed turtle, *Erymnochelys madagascariensis* across captive and wild populations

**DOI:** 10.1038/s41598-022-12422-y

**Published:** 2022-05-24

**Authors:** Nina F. D. White, Holly Mennell, Georgia Power, Dominic Edwards, Luke Chrimes, Lance Woolaver, Juliette Velosoa, Randriamahita, Richard Mozavelo, Tsilavo Hasina Rafeliarisoa, Gerald Kuchling, Javier Lopez, Ernest Bekarany, Namotoa Charles, Richard Young, Richard Lewis, Michael W. Bruford, Pablo Orozco-terWengel

**Affiliations:** 1 School of Biosciences, Cardiff University, Cardiff, UK; 2 Institute of Zoology, Zoological Society of London, London, UK; 3 Durrell Wildlife Conservation Trust, Les Augrès Manor, UK; 4 Wildlife Preservation Canada, Guelph, Canada; 5 Biodiversity Conservation Madagascar, Antananarivo, Madagascar; 6 School of Biological Sciences, University of Western Australia, Perth, Australia; 7 Animal Health Department, Chester Zoo, Cheshire, UK

**Keywords:** Conservation biology, Ecological genetics, Molecular ecology

## Abstract

*Erymnochelys madagascariensis* is a Critically Endangered turtle endemic to Madagascar. Anthropogenic activity has depleted the wild population by 70% in the last century, and effective conservation management is essential to ensuring its persistence. Captive breeding was implemented to augment depleted populations in the southern part of Ankarafantsika National Park (ANP), when no genetic data were available for *E. madagascariensis*. It is unknown how much of the natural population’s diversity is encapsulated in captivity. We used eight microsatellite loci and fragments of two mitochondrial genes to identify the genetic structure of *E. madagascariensis* in the wild. Captive bred turtles were compared with wild populations in order to assess the representativeness of this ex situ conservation strategy for ANP. Six microsatellite clusters, ten cytochrome b, and nine COI haplotypes were identified across wild populations, with high genetic divergence found between populations in two groups of watersheds. Captive bred individuals represent three out of six sampled microsatellite clusters found in the wild and just one mitochondrial haplotype, possibly due to genetic drift. To improve genetic representation, the strategy of frequent interchange between captive and wild breeders within ANP should be revitalised and, as originally planned, hatchlings or juveniles should not be released beyond ANP.

## Introduction

Madagascar is an island, separated from nearby land for many millions of years^1^. Madagascar’s separation from Africa occurred over 150 million years ago, and its most recent split occurred with India ~ 90 million years ago^1^. As a result, Madagascar’s biota has undergone unique evolutionary pathways in isolation and is largely endemic^2^. In an analysis of 25 regions with especially high levels of endemism endangered by habitat loss—‘biodiversity hotspots’—Madagascar is listed as the ‘hottest hotspot’ of all^3^. Malagasy biota is also characterised by ‘micro endemism’, meaning that many of the species are only found across a small geographic range within Madagascar^4^. Madagascar’s biodiversity is severely threatened by anthropogenic activity: agriculture, deforestation, illegal poaching and the wildlife trade, and the introduction of non-native species^5^. Of the 327 reptile species on the island, 92% are endemic and 39% of these are at risk of extinction^3,6^.

One such threatened endemic reptile is the Critically Endangered Madagascar big-headed turtle *Erymnochelys madagascariensis*, the Rere, or Madagascar side-necked turtle^7,8^. *Erymnochelys madagascariensis* is the only member of the *Erymnochelys* genus and also the only existing old world member of the family Podocnemididae^9,10^. *Erymnochelys madagascariensis* is endemic to western Madagascar^7^. An estimated 10,000 individuals are distributed in subpopulations along a stretch of eight lowland watersheds^7,8^. The species is mainly sedentary but individuals will migrate into seasonally flooded areas to find better sources of food, or in dry conditions to reach new refuge wetlands^11^. Being limited to a specific freshwater aquatic habitat, *E. madagascariensis* is vulnerable to anthropogenic disturbance in this type of environment^8^, for example, when wetlands are drained for conversion to rice paddies^12^. Of all the endemic Malagasy chelonians, *E. madagascariensis* has the oldest age of sexual maturity at 18–25 years old making the species especially susceptible to population decline if individuals are removed from the population before reaching maturity^13^. Illegal trade and consumption of Reres and their eggs is a major threat to the survival of *E. madagascariensis*. Turtles may reach ~ 26 cm in length before they are able to breed and due to their large size, they are an attractive food source even when sexually immature^8,13^. Additionally, Rere habitat loss and degradation is being precipitated by deforestation, soil erosion and river siltation^14^. As a result of human activity, *E. madagascariensis* is now only present in 7.6% of its historical geographical range, and on the verge of extinction in a quarter of this remaining area^7^.

IUCN classified *E. madagascariensis* as Endangered in 1996 and conservation of the species began in 1998^8^. The species was reclassified as Critically Endangered in 2008^15^. The long-term goal for *E. madagascariensis* conservation is to have at least one viable population in each of the eight watersheds inhabited across the turtle’s entire range^7^. Conservation involves research and monitoring of wild populations, the engagement of local communities, population management through captive breeding, and head starting of juveniles between populations^7,16^. Hatchlings collected from wild nests in Ankarafantsika National Park (ANP) are head started for three to ten years in the captive breeding facility in Ampijoroa (set up in 1999) in parallel with captive bred hatchlings prior to release into the wild to augment depleted populations at ANP^7^. To date, captive breeding in this facility has contributed 114 hatchlings to the population of *E. madagascariensis* at lake Ravelobe (J. Velosoa, *pers. comm*.). However, at the time of the facility’s start in 1999, no information existed on the genetic structure of *E. madagascariensis*^7^. As the population genetic structure was unknown, a representative sample from the whole range of *E. madagascariensis*—as recommended when starting ex situ breeding programmes for the long-term securing of a species in captivity^17^—was not used in the ex situ strategy, as this could risk the loss of unique genetic variation that may have existed within different isolated subpopulations. As a result, the captive population was created from nine turtles within ~ 30 km of the southern part of Ankarafantsika National Park (ANP) to conserve any local genetic diversity from turtles in that area^7^. Analysing the genetics of founder populations before the initiation of ex situ conservation breeding is recommended to avoid the loss of genetic diversity^17^, but is not always possible, and many conservation programs now face the task of reconstructing the genetic history and representativeness of captive populations post hoc in order to better manage genetic diversity in the present population and for future viability, see^18^. Little published information exists on the genetic structure of *E. madagascariensis* in the wild. Velosoa *et al*.^7^ mention an unpublished report which describes a divergence between northern and southern subpopulations, and a potential but as yet unidentified hybrid zone. In light of this, Velosoa *et al*. recommend that future research focus on understanding the genetic structure that exists across the range of *E. madagascariensis*. It is important to provide a published description of the genetic structure of *E. madagascariensis*, based on clearly defined sampling, locations, and genetic markers.

Rivers have been shown to act as barriers to gene flow in several Malagasy reptiles including tree boas, geckos and chameleons^19^. In all cases, genetically distinct subpopulations have arisen either side of rivers, as a result of the reptiles’ inability to cross water. *Erymnochelys madagascariensis*, however, is in principle, easily able to move along and across waterways, although it is unclear as to what extent it does so in practice. An example of a water-dependent species that also exhibits genetic differentiation due to bodies of water in Madagascar is the tomato frog *Dyscophus guineti*, in which genetic variation arises due to its limited dispersal beyond rivers^20^. If water-dependent amphibians with low vagility in Madagascar exhibit genetic differentiation in different geographical areas^20^, it is possible that water-dependent reptiles known to be largely sedentary may also exhibit divergent genotypes at different geographically isolated areas—i.e. different watersheds. Similarly, a phenomenon originally described by Wilmé *et al*.^2^ and termed the ‘watershed hypothesis’ by Pearson and Raxworthy^21^ suggests that watersheds, combined with historic climatic shifts, drive patterns of endemism and speciation in Madagascar. Wilmé *et al*.^2^ describe ‘retreat-dispersion watersheds’ (RDW) which maintained cooler, moister climates at higher elevations during periods of lowland aridity. The higher elevations of RDWs may have served as a retreat from inhospitable lowlands during the Quaternary. The initial retreat of some populations, coupled with their eventual dispersal back into lower elevations, may have served to isolate populations and drive allopatric speciation^21^. Indeed, the position of RDWs significantly correlate with the distribution of many species in Madagascar^21^. Understanding the genetic variation across the range of wild turtles may help to direct conservation goals towards maintaining specific levels and/or elements of diversity in the wild (e.g. avoid inbreeding). Secondly, this information may help conservationists to better implement the full range of genetic variation in additional captive populations, and in doing so decrease the risk of inbreeding.

Here we present the results from an analysis of genetic variation in 467 *E. madagascariensis* individuals from across the species’ range in north-western Madagascar, using eight microsatellite markers and partial sequences for the mitochondrial genes cytochrome b and cytochrome oxidase subunit I (COI). These data were used to analyse *E. madagascariensis* samples from 22 locations and test the hypothesis that genetic structure exists between different watersheds. We also assessed the genetic variation between captive bred individuals and wild individuals, to establish the genetic representation of the captive population. As the ex situ operation has always been limited to providing translocation stock to augment depleted populations in the southern part of ANP, we hypothesise that this captive population would only be genetically representative of ANP, rather than all of the wild diversity available throughout the range of the species. Our findings are then contextualised within their possible implications for future conservation of *E. madagascariensis*.

## Methods

### Sample collection and DNA extraction

**Figure 1:**
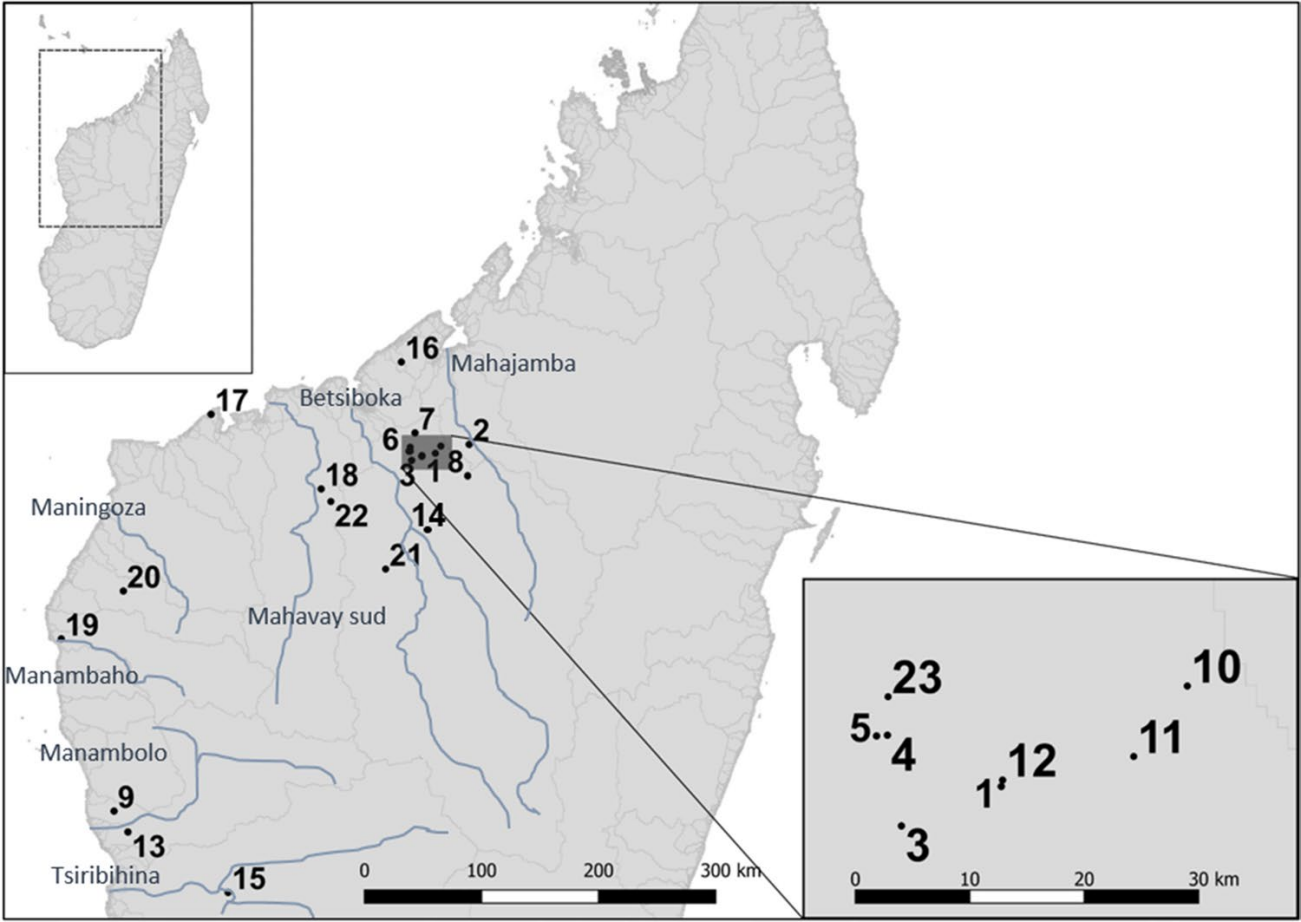
Locations of *E. madagascariensis* sample collection. Madagascar shown in the top left insert, with general sampling area outlined. Specific sampling locations are numbered on the main map, and refer to: (1) Captive facility (Ampijoroa), (2) Andranomiditra, (3) Ankomakoma, (4) Ankorovoka, (5) Antsilomba, (6) Bemangaoka, (7) Amboromalandy* (8) Kamoro*, (9) Ankerika, (10) Antsiloky, (11) Matsaborimavo, (12) Ravelobe, (13) Ambondrobe, (14) Amparihibe, (15) Ankazomanga, (16) Marovoay Kely, (17) Sariaka, (18) Mahavay-Sud, (19) Manambaho, (20) Ankilolio, (21) Ikopa, (22) Sitampiky, (23) Vavanimarovoay. The highlighted area is Ankarafantsika National Park. Grey lines on the map indicate watershed borders, blue lines and text are rivers and their names. * indicates a location that a sample was confiscated from. Map generated in QGIS v.3.4 (http://www.qgis.org).

A total of 467 blood or tissue samples were taken from individuals across 23 locations, and seven watersheds across north-western Madagascar (Fig. 1). Tissue samples were 2 mm foot web clippings and blood was collected from the jugular vein. Three hundred and seventy-eight wild samples were taken between 2002 and 2015. Ninety captive bred hatchlings were sampled between 2004 and 2015 from the captive breeding facility at Ampijoroa. All samples were stored in 70–100% ethanol. DNA was isolated using the QIAGEN DNeasy Animal Blood and Tissue Kit. Step two of the Qiagen protocol was modified so that samples were incubated at 37 °C for 14–17 h.

### Genotyping

Genotyping was carried out using eight microsatellite markers and two mitochondrial gene fragments. While population genetic studies are beginning to favour high-throughput sequencing based methods such as RAD-seq, these markers were chosen due to the availability of pre-existing primers, which minimised labour and financial constraints on the project. Microsatellites, especially with a large sample size and supported by mtDNA, can produce genetic diversity and structure results in line with genomic methods (e.g. RAD-seq)^22^. Microsatellite primers^9^ (Supplementary Table S1) were arranged into multiplex reactions using 1 µL of DNA, 5 µL of QIAGEN Multiplex solution and 0.1 µL of each primer. The thermal profile for PCR amplification consisted of an initial denaturation of 95 °C for 15 min, followed by 35 cycles of 94 °C for 1 min, variable temperatures (Supplementary Table S1) for 1 min, 72 °C for 1 min, and a final extension of 72 °C for 10 min. Samples were diluted 1 in 2 for fragment analysis carried out by DNA Sequencing and Services, University of Dundee using a ROX 500 standard size marker. Resulting alleles were scored using GeneMarker v1.91^23^. Microchecker^24^ was used to check for the presence of null alleles in the data. Null alleles detected in populations with fewer than 15 individuals or at frequencies lower than 8% were disregarded, as they are unlikely to have significant effects on population assignment^25^.

A 342 bp fragment of the cytochrome b gene primers was produced using the following primer sequences: Forward (CB-J-10933) = 5′-TATGTTCTACCATGAGGACAAATATC-3′, Reverse (CytbC) = 5′-CTACTGGTTGTCCTCCGATTCATGT-3′^26^. DNA amplification was conducted using 5 µL of QIAGEN Multiplex solution, 3.8 µL of water and 0.1 µL of each primer, per 1 µL of DNA. The PCR thermal profile consisted of an initial denaturation of 95 °C for 15 min, followed by 30 cycles of 94 °C for 30 s, 55 °C for 30 s, 72 °C for 30 s, with a final extension of 72 °C for 10 min. Cytochrome oxidase subunit 1 (COI) primers used to amplify a 300 bp fragment were as follows: M72L (5′-TGATTCTTCGGTCACCCAGAAGTGTA -3′) M73H (5′-CCTATTGATAGGACGTAGTGGAAG -3′). PCR reaction volumes of 20 µL were used consisting of: 13.58 µL H_2_O, 2.5 µL 5 × Go Taq Flexi Buffer, 0.2 µL 25 Mm MgCl_2_, 0.32 µL 10 p/mol dNTPs, 0.2 µL Go Taq Flexi DNA polymerase, 0.2 µL of each primer and 1 µL of DNA. PCR conditions consisted of 40 cycles of an initial 95 °C for 15 min, denaturation at 94 °C for 1 min, annealing at 54 °C for 1 min, extension at 72 °C for 1 min, and final extension at 72 °C for 10 min. PCR products were purified using 1.25 µL of 10 × SAP buffer, 0.5 µL of Thermosensitive Alkaline Phosphate and 0.25 µL of Exonuclease I per 10 µL of PCR product. The purified product was then sequenced by Eurofins Genomics.

### Analysis of demography

To aid in the interpretation of genetic structure and diversity results, the demographic history of the Rere was investigated. Linked microsatellite loci can be an indication of non-random mating in populations, and Arlequin was used to perform tests for linkage disequilibrium between microsatellite locus pairs. To assess the presence and extent of gene flow between sampling locations, the number of migrants (Nm) based on the private allele method was estimated in Genepop^27^. To understand how the Rere’s population size has changed throughout history, an analysis of demography was carried out. Sampling locations with n ≥ 10 were analysed for evidence of a genetic bottleneck, population expansion or stability using Msvar v1.3^28^. For sampling locations with n ≥ 20, 20 individuals were randomly subsampled for the analysis to restrict the computation time. A standard vertebrate mutation rate was used (10^−3^–10^−5^) as no specific value was available for *E. madagascariensis*^29^. Generation time was set at 25 years^13^. 4 × 10^9^ iterations were performed with 20% discarded as burn in. A Gelman & Rubin test^30^ from the CODA library^31^ was used to identify convergence of runs under three different prior scenarios in R statistical software^32^. Priors assumed a stable population, a population expansion, and a population bottleneck, allowing confirmation that the posterior distributions resulting from Msvar analysis were not biased by these priors. MtDNA samples were not included in demography analysis, because they only partially represent the whole dataset, and mtDNA can only give an insight to maternal lineage demography.

### Analysis of genetic structure

To examine how many distinct ‘clusters’ of individuals with similar genetic variation exist within the dataset, and whether these clusters associate with sampling locations, a Structure (v.2.3.4) analysis was run^33^. To avoid biasing the determination of genetic clusters with missing data, all microsatellite samples with missing data for ≥ 1 loci were removed from the dataset (~ 5% of the total set of samples was removed for this analysis). Similarly, to minimise the chance of incorrectly inferring K (number of genetic clusters), the magnitude of uneven sampling in the dataset was reduced^34^. This was achieved by randomly subsampling all populations of n ≥ 30, without replacement, to include only 30 individuals (deemed appropriate by^35^), before performing Structure analysis. Three different resampled datasets were analysed to confirm that 30 was an appropriate number, and that differently subsampled populations did not generate largely different results in Structure. Structure was run using 500,000 MCMC iterations with 100,000 discarded as burn in and K set between 1 and 8. Three iterations of each analysis were run to assess convergence between results. POPHELPER Structure Web App v.1.0.10^36^ was used to generate bar plots from Structure results. To determine the most likely K, three methods were used. Two Evanno plots were used—Mean L(K) and ΔK^37^. Especially in the instance of uneven sampling, inaccuracies in the estimation of K using Evanno methods have been identified^34^. Consequently, a third, independent set of methods was used and calculated manually—termed MedMeaK, MaxMeaK, MedMedK and MaxMedK (MMMM test)^34^. A threshold of 0.8 was used as the most stringent estimator of K. Consensus sequences for mtDNA were produced in Geneious v.6.06^38^. MEGA was used to create Maximum Likelihood (ML) trees for cytochrome b and COI with a bootstrap value of 100 for sampling locations and haplotypes.

### Analysis of genetic diversity

Summary statistics were calculated to estimate the populations’ genetic variation and divergence. We used Microsatellite Analyser v4.05^39^ to estimate the observed and expected heterozygosity, allelic richness, average number of alleles per locus, and pairwise F_ST_. This was performed using the full microsatellite dataset, grouped according to sampling localities and by genetic cluster. For F_ST_ significance was assessed with 10,000 permutations and applying Benjamini and Yekutieli false discovery rate (FDR) correction^40^. Inbreeding coefficient (F_IS_) values and significance were calculated using FSTAT v2.9.3^41^. To assess the partition of genetic diversity within and between sampling locations, genetic clusters, or phylogenetic clade (for mtDNA), an AMOVA was performed in Arlequin v3.5^42^ using 1000 permutations. If genetic clusters or clades can better distinguish genetic lineages than individual sampling locations, this is important to guide conservation management. Genepop v4.6^27^ was used to perform an exact test to determine whether the sampling locations and genetic clusters were in Hardy–Weinberg equilibrium. As the standard errors of the Genepop results were small (less than the order of P) and the number of switches high (> 1000), the Markov chain run parameters were suitable and left as default^27^. Heterozygote deficiency and excess were also calculated to explain any deviation from the Hardy–Weinberg equilibrium. To compare mtDNA diversity between populations, the number and diversity of haplotypes for both cytochrome b and COI were calculated using DNAsp v5.10.1^43^. In order to compare the genetic representativeness of the captive population with the genetic diversity expected from a wild population of the same size, a bootstrap resampling method was employed for microsatellite and mtDNA. One hundred random datasets of 47 individuals (the same number of COI sequences available for individuals from the captive population) were generated from the 67 wild individuals with available COI sequences. The same was done for cytochrome b, resampling the 50 wild individuals in sets of six. Haplotype diversity and pi, generated using DNAsp for all 100 datasets and the captive population, were used to create expected distribution plots for the wild population’s mtDNA diversity distribution compared to the captive population. The same process was repeated for microsatellite data, resampling the 354 wild individuals to replicate the captive sample size of 88 individuals. Distributions of heterozygosity and average number of alleles per locus were generated. TempNet^44^ was used to illustrate the distribution of mitochondrial haplotypes between the overall wild population and the captive bred hatchlings.

### Ethics statement

Sampling was carried out by a veterinarian according to blood and tissue sampling protocols approved by Durrell Wildlife Conservation Trust and the Madagascar Ministry of the Environment, Ecology, Sea and Forests (MEEMF) under permit number M6/15/MEEMF/SG/DGF/DCB.SAPT/SCBT. All samples were exported with CITES permit no. 617C-EA07/MG15 and imported with CITES permit nos. 537437/01, 537437/02, 537437/03.

## Results

Twenty-six samples were discarded due to missing data, leaving 442 samples for the remaining microsatellite analyses. Null alleles were detected at two loci in sampling location populations with more than 15 individuals or at a frequency > 0.8%. T-tests revealed no significant effect on heterozygosity or F_ST_ when null alleles were removed from the analysis (*p* value > 0.05). Subsequently no loci were excluded due to the presence of null alleles. Sequences of sufficient quality were produced for 56 individuals for cytochrome b, and 114 for COI.

### Demography

**Figure 2:**
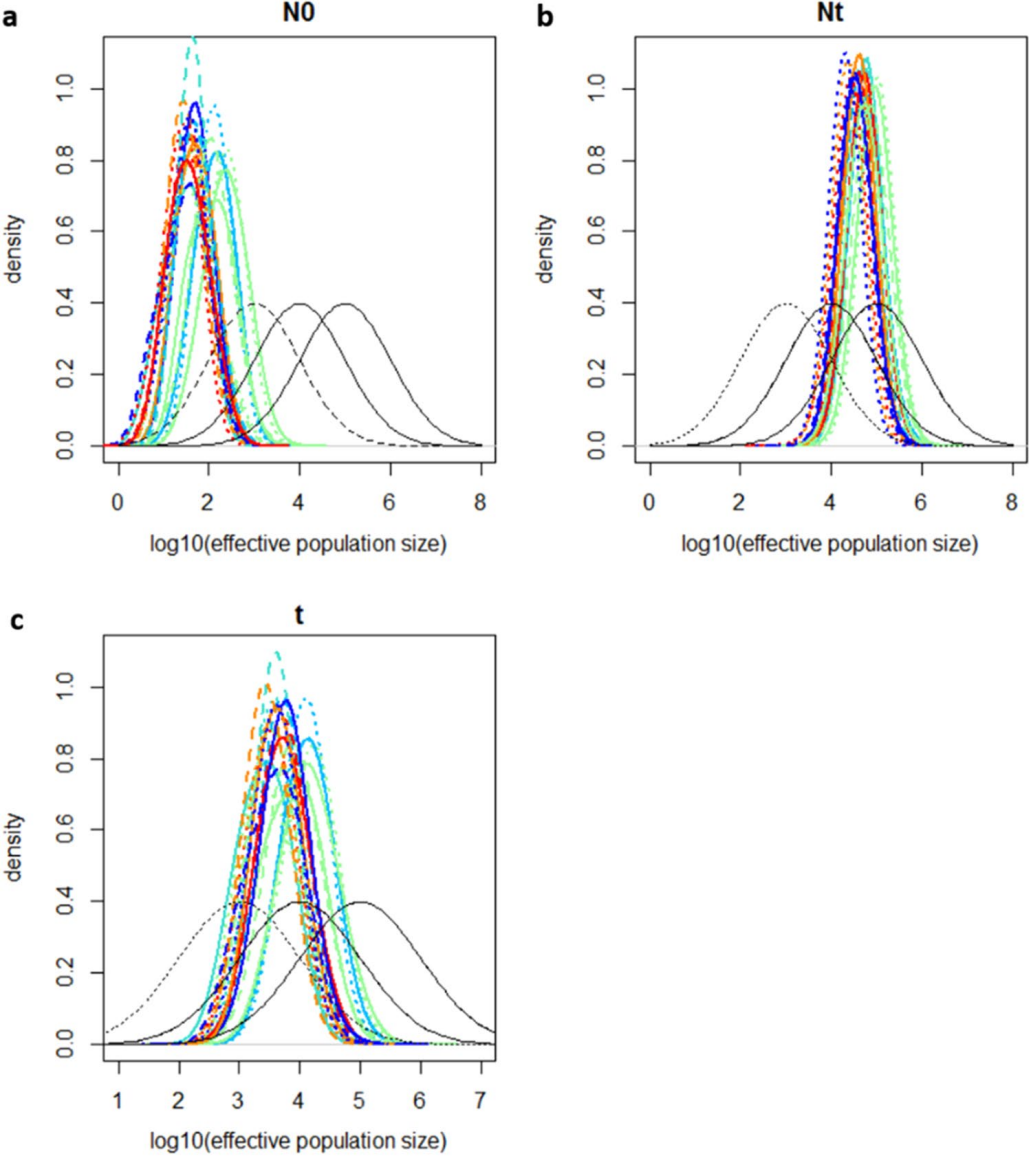
Posterior distributions of the parameters Nt (ancestral population size—**a**), N0 (current effective population size—**b**) and t (time of bottleneck—**c**), produced in Msvar. For all plots, coloured lines represent populations. Black lines on plots represent prior distributions: solid = stable, dashed = expansion, dotted = bottleneck.

Only the captive bred population had loci which were not independent of each other, with 13 out of 28 pairs exhibiting linkage disequilibrium (FDR corrected *p* value < 0.05). More private alleles were detected amongst sampling locations (0.065) than genetic clusters (0.02). When corrected for sample size, 1.79 migrants per generation were detected between sampling locations, compared to 4.24 between genetic clusters. With a mean generation time of ~ 40 years, this equates to ~ 9 individuals moving between locations over 200 years. To search for demographic processes that may underlie some of these observations, an Msvar analysis was performed. Three different combinations of priors were used and the convergence of the Msvar MCMC was determined using Gelman & Rubin statistics that were < 1.2 (indicating convergence of Msvar runs). Priors and hyperpriors used are shown in Supplementary Table S2. Modes and 95% higher poster density intervals for all priors are in Supplementary Table S3. All populations were observed to have passed through a genetic bottleneck (Nt > N0) at ~ 5600 years ago, with an average lower and highest posterior density interval of 549–14,791 years ago (Fig. 2). In support of a historic population-wide bottleneck, Nt/N0 ≥ 1 for every population.

### Genetic structure

**Figure 3:**
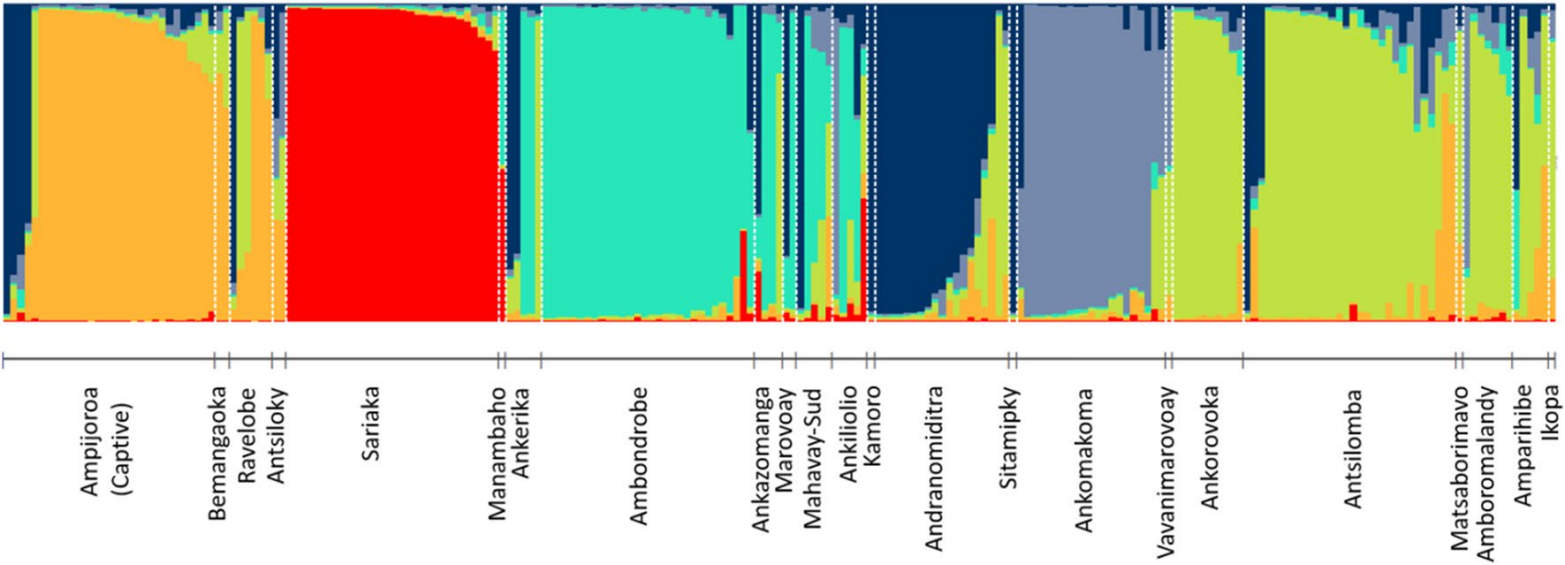
Membership of individuals from each sampling location to genetic clusters determined using Bayesian cluster analysis with K = 6. The captive population is labelled, and all other populations are wild.

**Figure 4:**
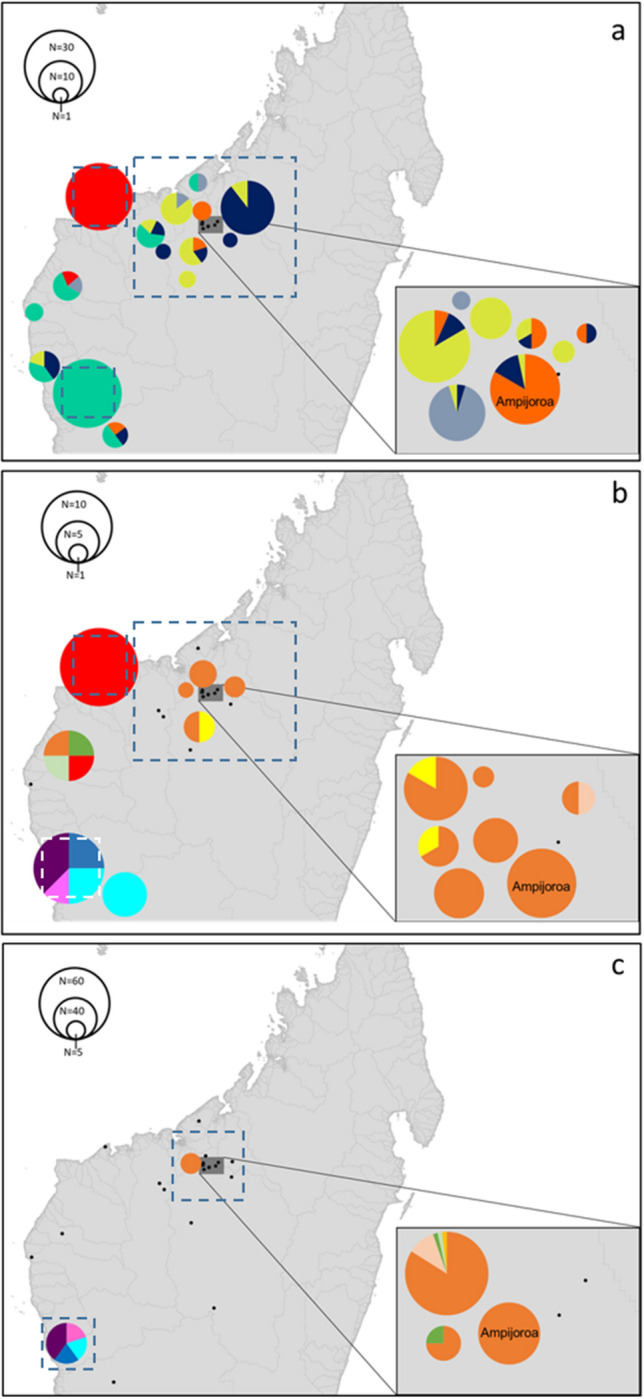
Comparative distribution of genetic structure across the sampled area for (**a**) microsatellite genetic cluster, (**b**) cytochrome b, and (**c**) cytochrome oxidase 1 mitochondrial haplotypes. Size of circle corresponds to population size. Putative management units for conservation are indicated by dashed rectangles. Maps generated in QGIS v.3.4 (http://www.qgis.org)

Based on Evanno and MMMM methods, the most likely number of genetic clusters was six (Fig. 3). Captive bred hatchlings formed a genetic cluster with individuals from Lakes Ravelobe and Antsiloky, two of three founder locations used for the captive population. F_ST_ values between clusters ranged from 0.01 (cluster 2–3) to 0.30 (cluster 3–5) and were all significant (Table 1). Some geographical restriction of genetic structure was observed. Cluster 2 was restricted to the north-west of the sampling area, in Lake Sariaka and Ankilolio (Fig. 4). Cluster 3 was dominant in the Tsiribihina, Manambolo, Manambaho, and Maningoza watersheds, and to a lesser extent in Mahavay sud, but not found in the Betsiboka watershed (Fig. 4). All other clusters were observed to occur in more than one watershed of the sampling area. The most microsatellite variation (81%) originated within sampling locations based on AMOVA analysis (Supplementary Table S4). Sampling locations within genetic clusters gave the smallest variance (6%), consistent with the grouping of locations with Structure. All pairwise F_ST_ comparisons were significant between sampling locations with n > 10 (Table 1). The captive bred population at Ampijoroa was most diverged from populations at Lakes Sariaka and Ambondrobe (Table 1). All locations with n > 1 had a mixture of genetic clusters, except Ambondrobe, Sariaka and Ankorovoka in which all individuals belonged to one genetic cluster.

**Table 1:** Above diagonal = pairwise F_ST_ values; below = false discovery rate corrected *p* values (Benjamini and Yetukieli method).

Sampling locations	Ampijoroa	Andranomiditra	Ankomakoma	Ankorovoka	Antsilomba	Ambondrobe	Sariaka
Ampijoroa	–	0.12	0.12	0.10	0.14	0.21	0.32
Andranomiditra	0.0004	–	0.16	0.07	0.06	0.22	0.37
Ankomakoma	0.0004	0.0004	–	0.10	0.14	0.19	0.35
Ankorovoka	0.0004	0.0015	0.0004	–	0.02	0.18	0.36
Antsilomba	0.0004	0.0004	0.0004	0.035	–	0.21	0.33
Ambondrobe	0.0004	0.0004	0.0004	0.0004	0.0004	–	0.27
Sariaka	0.0004	0.0004	0.0004	0.0004	0.0004	0.0004	–

Genetic clusters	Cluster 1	Cluster 2	Cluster 3	Cluster 4	Cluster 5	Cluster 6	
Cluster 1	–	0.08	0.09	0.06	0.21	0.07	
Cluster 2	0.0004	–	0.01	0.12	0.29	0.06	
Cluster 3	0.0004	0.03	–	0.13	0.30	0.06	
Cluster 4	0.0004	0.0004	0.0004	–	0.08	0.10	
Cluster 5	0.0004	0.0004	0.0004	0.0004	–	0.23	
Cluster 6	0.0004	0.0004	0.0004	0.0004	0.0004	–

The cytochrome b ML tree revealed three major clades, separating the Mahajamba and Betsiboka watersheds into one clade, and all watersheds into another clade with a bootstrap support of 99 (Supplementary Fig. S1). The further separation of Sariaka and Ankilolio from Ambondrobe and Ankazomanga in the more south-westerly Manambolo and Tsiribihina watersheds, was supported by bootstrap values of 99% and 96%. A ML tree of haplotypes reflected this trend. COI data supported the separation of Lake Ambondrobe with a bootstrap value of 100 (Supplementary Fig. S2). From a haplotype ML tree, haplotypes 6–9 belonging to Ambondrobe separated from other populations with a bootstrap support of 100 (Supplementary Fig. S2). These trends can also be seen in Fig. 4. An AMOVA revealed 93% of variance in cytochrome b haplotypes between phylogenetic clades—which corresponded to the groups of watersheds mentioned above (Supplementary Table S4). The lowest variance was found between populations within clades (0.1%), and the final 7% was found within populations. The same pattern was observed for COI, with 95% of variation originating between phylogenetic clades, 0.35% among populations in the same clade and 4% between populations (Supplementary Table S4).

### Genetic diversity

The average heterozygosity observed across wild sampling locations ranged from 0.5 to 1 (Table 2). The captive bred hatchlings at Ampijoroa had higher than expected heterozygosity and significant outbreeding based on F_IS_ (Table 2). Average allelic richness ranged from 1.5 to 2 in the wild metapopulation and was 1.71 in the captive population (Table 2). There were 643 alleles across wild locations, compared with 54 in the captive population. When averaged across loci, the average number of alleles was 1.5 times higher in the wild population (treated as one population) than the captive (Table 2). No significant difference in heterozygosity or allelic richness between the captive and overall wild population was detected using a t-test (*p* values = 0.069, 0.23, respectively). Cluster 6 had the highest average observed heterozygosity while cluster 5 had the lowest (Table 2). All clusters had lower than expected heterozygosity. Average allelic richness was highest in cluster 4 and lowest in cluster 5 (Table 2). The captive population had the highest number of loci with heterozygote excess (Table 2). A significant level of inbreeding was detected in all clusters besides number 5 (Table 2). No genetic clusters were observed to be in Hardy–Weinberg equilibrium (Fisher’s method χ^2^ = infinity, df = 48, prob = highly significant). The highest observed heterozygosity amongst the wild locations with n > 1 was found at Bemangaoka (n = 2). The highest heterozygosity in a location with > 5 individuals was at Amboromalandy (Table 2). Sariaka had the lowest heterozygosity and number of alleles. The largest number of alleles, accounting for sample size, was found at Amparihibe, though a significant level of inbreeding was also detected here (Table 2). Antsilomba was the only other population to have a significant inbreeding coefficient (Table 2).

**Table 2:** Genetic summary statistics, averaged for captive and wild populations, and genetic clusters.

Population	N	H_O_	H_E_	A_R_	A_n_	F_IS_	HEx	HDe
Captive	88	0.78	0.71	1.71	6.75	− 0.10*	3	1
Wild	354	0.66	0.66	1.66	10.05	0.05	0	2
Cluster 1	98	0.61	0.71	7.05	9.88	0.14*	0	7
Cluster 2	41	0.58	0.64	6.48	7.63	0.10*	0	3
Cluster 3	77	0.58	0.63	6.35	8.75	0.08*	0	5
Cluster 4	21	0.61	0.73	7.50	7.50	0.16*	1	4
Cluster 5	22	0.52	0.55	4.81	4.88	0.04	0	3
Cluster 6	183	0.68	0.75	7.26	10.88	0.09*	0	7
Ampijoroa	88	0.78	0.71	1.71	6.75	− 0.10*	3	1
Andranomiditra	19	0.66	0.62	1.62	4.38	− 0.07	2	2
Ankomakoma	21	0.60	0.64	1.64	4.88	0.06	0	0
Ankorovoka	10	0.59	0.68	1.68	4.75	0.14	0	2
Antsilomba	159	0.57	0.62	1.62	9.88	0.08*	0	7
Bemangaoka	2	0.88	0.71	1.71	2.38	− 0.40	0	0
Amboromalandy	7	0.73	0.62	1.62	4.25	− 0.21	1	0
Kamoro	1	0.50	0.50	1.50	1.50	NA	–	–
Ankerika	5	0.60	0.62	1.62	3.13	0.04	0	0
Antsiloky	2	0.75	0.67	1.67	2.50	− 0.20	0	0
Matsaborimavo	1	0.75	0.75	1.75	1.75	NA	–	–
Ravelobe	6	0.73	0.67	1.67	3.63	− 0.10	0	0
Ambondrobe	56	0.60	0.64	1.64	7.13	0.07	0	4
Amparihibe	5	0.53	0.72	1.72	4.50	0.29*	0	1
Ankazomanga	4	0.63	0.69	1.69	3.63	0.11	0	0
Marovoay	2	0.63	0.60	1.60	2.38	− 0.05	0	0
Sariaka	40	0.51	0.51	1.51	4.63	0.00	1	0
Mahavay-Sud	5	0.65	0.70	1.70	4.50	0.08	0	3
Manambaho	1	0.75	0.75	1.75	1.75	NA	–	–
Ankiliolio	5	0.73	0.68	1.68	3.75	− 0.07	0	1
Ikopa	1	1.00	1.00	2.00	2.00	NA	–	–
Sitamipky	1	0.50	0.50	1.50	1.50	NA	–	–
Vavanimarovoay	1	0.63	0.63	1.63	1.63	NA	–	–

*H*_*O*_ observed heterozygosity, *H*_*E*_ expected heterozygosity, *A*_*R*_ allelic richness, *A*_*n*_ average number of alleles per locus, *F*_*IS*_ inbreeding coefficient (* indicates a false discovery rate corrected *p* value < 0.05), *HEx* no. loci with a significant heterozygote excess, *HDe* no. loci with a significant heterozygote deficiency.

**Figure 5:**
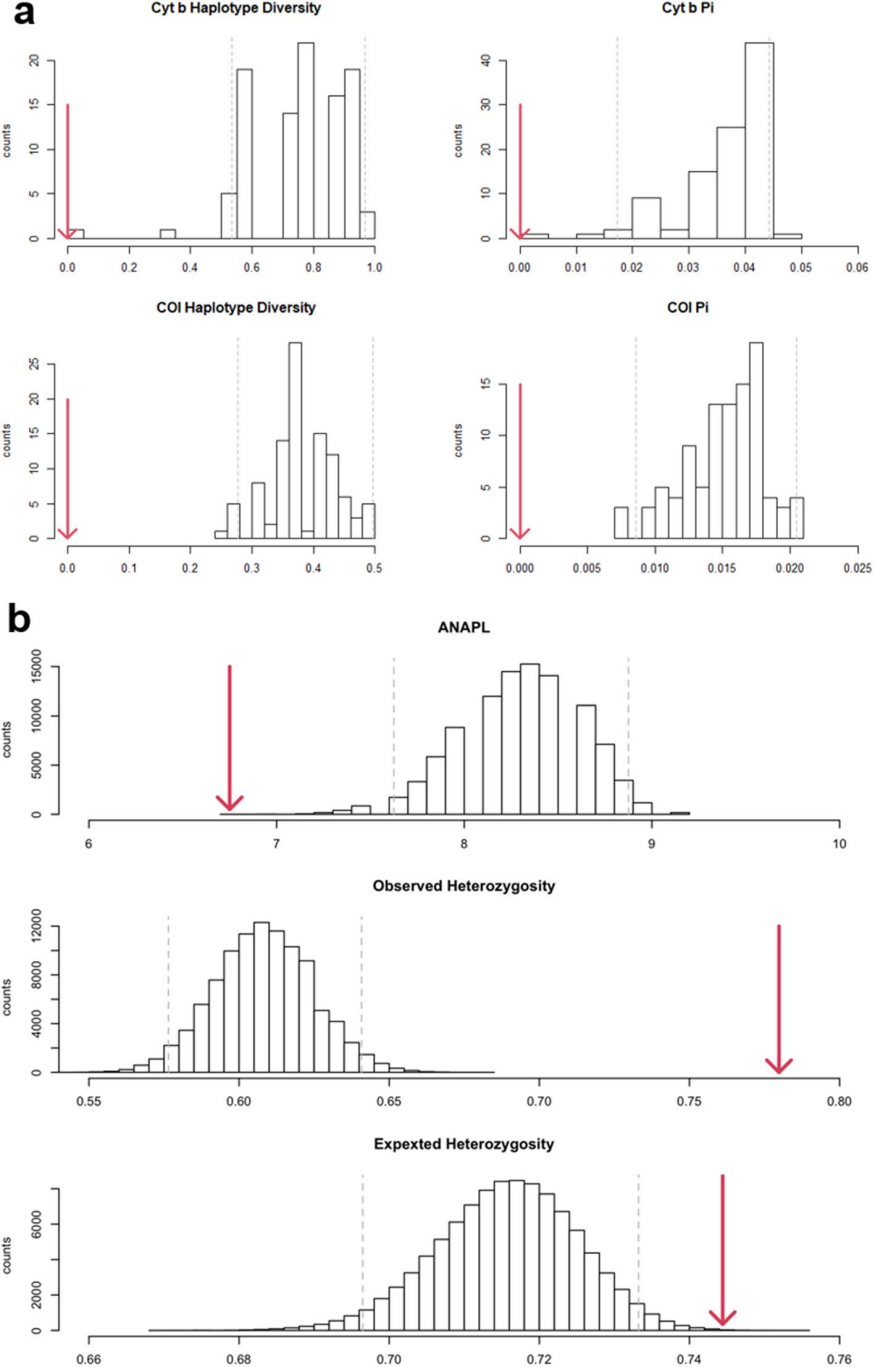
Expected distribution of genetic variation in the wild metapopulation for (**a**) mitochondrial, and (**b**) microsatellites. The red line indicates the value for the captive population. *Pi* nucleotide diversity, *ANAPL* average number of alleles per locus.

**Figure 6:**
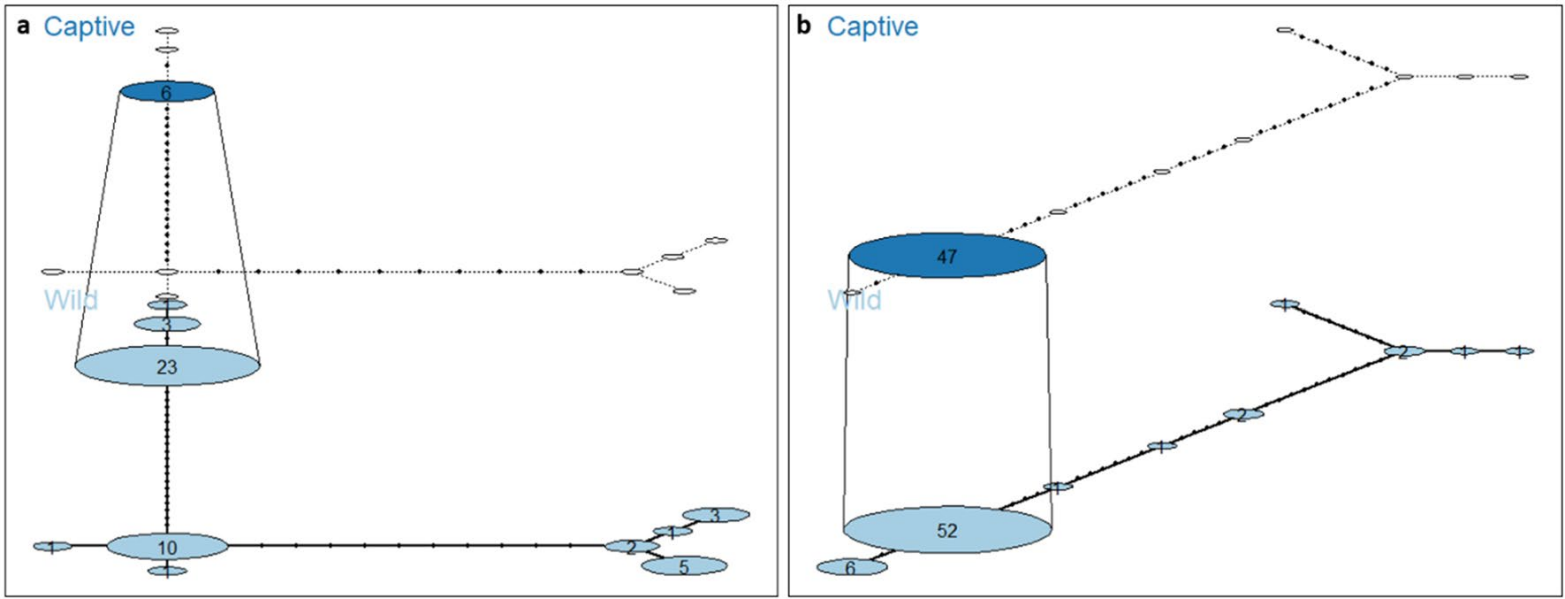
TempNet Network depicting the mitochondrial haplotypes for (**a**) cytochrome B, and (**b**) cytochrome oxidase I sequences, found in the total wild population of *E. madagascariensis* and in the captive bred population at Ampijoroa. Each population is shown on a different layer of the network, and shared haplotypes between populations are joined with vertical lines. Filled, blue ellipses represent sampled haplotypes, and are sized and labelled with the number of samples belonging to the haplotype. Unfilled ellipses represent unsampled haplotypes in a population. Small, black circles represent unsampled haplotypes across all populations. Dotted lines represent connections containing missing haplotypes.

From 56 cytochrome b sequences across 15 sampling locations, 10 haplotypes were found. Haplotype diversity was 0.696 (var = 0.0033, SD = 0.058) and Pi 0.034. COI data revealed 9 haplotypes present across five locations from 114 sequences. Lower COI haplotype diversity of 0.244 (var = 0.0028, SD = 0.053) was observed, along with by Pi = 0.0095. The captive bred population from Ampijoroa was represented by one haplotype for each mitochondrial marker (Figs. 4 and 6). The highest cytochrome b diversity was found at Ankilolio and Ambondrobe (four haplotypes), and at Antsilomba for COI (five haplotypes). Mitochondrial data revealed that the Reres bred at Ampijoroa are not genetically representative of the wild metapopulation, with both haplotype diversity and nucleotide diversity (Pi) below the 2.5% confidence interval for the expected genetic variation of the wild metapopulation (Fig. 5). Microsatellite data revealed observed and expected heterozygosity in the captive population lay above the 97.5% confidence interval for the wild metapopulation. The average number of alleles per locus in the captive population was lower than the 2.5% confidence interval for the expected distribution of the wild population taken as a whole (Fig. 5). This is illustrated in Fig. 6, a TempNet network showing just one haplotype shared with the wild in the captive bred population.

## Discussion

This study has identified key divisions of genetic variation and patterns of diversity across the sampled range *E. madagascariensis* which can inform future species management. Microsatellite and mitochondrial markers revealed similar geographic patterns underlying the genetic structure of *E. madagascariensis*. All methods identified genotypes that were restricted to the Mahajamba and Betsiboka watersheds surrounding ANP, and genotypes restricted to at least another watershed, further west. Lake Sariaka’s and Lake Ambondrobe’s populations were consistently identified as genetically distinct. Sariaka was characterised by a single microsatellite cluster or mitochondrial haplotype, while Ambondrobe and neighbouring Ankazomanga had unique haplotypes, not found in the watersheds east of Mahavay sud. Lakes Sariaka and Ambondrobe displayed the greatest genetic divergence from all other populations, with the largest averaged F_ST_ values (averaging across all pairwise comparisons with all other populations; 0.34 and 0.21, respectively) thus providing evidence of their genetic divergence. The geographic distance between Lakes Sariaka and Ambondrobe, and the population most genetically diverged from them (at Andranomiditra River) is ~ 300 km. The maximum F_ST_ values between populations of another Malagasy testudine (*Geochelone radiata)* over a similar range were smaller than those identified for populations of *E. madagascariensis*^45^. This suggests that individuals from Lake Sariaka exhibit a notably large genetic divergence with respect to other Rere populations. Furthermore, of the sampling locations with > 5 individuals, Lake Sariaka had the lowest heterozygosity (0.51) which may be indicative of genetic drift or inbreeding. Cytochrome b data revealed a divide between individuals in the most north-westerly point of the sampled area (Lake Sariaka and Akilolio), and the south-west of the sampling area (Manambolo and Tsiribihina watersheds). COI data supported this divide, as far as the limited data for this marker could show. As no data were collected for individuals in north-westerly Maningoza watershed or Lake Sariaka, the additional division between north and south westerly populations, as seen with cytochrome b data, cannot be assessed. Microsatellite data did not reveal such distinct geographical separation of genotypes, but cluster 2 was restricted just to Lake Sariaka.

Watershed boundaries can structure the genetic similarity between freshwater turtles, for example the Wood turtle^46^. Watersheds in Fig. 1 do not explain microsatellite population structure as strictly in the Rere. All clusters were found across more than one watershed. Contrastingly, mitochondrial haplotypes were often restricted to one watershed—however a third of cytochrome b and 95% of COI haplotypes originate in the same watershed as ANP, where sampling effort was highest and therefore more likely to pick up unique haplotypes. Higher differentiation in mitochondrial than microsatellite markers between watersheds could be due to fidelity to one river for breeding by female turtles—as observed in *Podocnemis expansa*^47,48^. As mitochondria are maternally inherited, female preference for one watershed could limit the spread of haplotypes outside of that watershed, in contrast to microsatellites which may be inherited from either sex^47,49^. *Erymnochelys madagascariensis’* genetic structure could be better explained by a retreat-dispersion watershed pattern, as suggested for many other patterns of genetic diversity found throughout Madagascar^21^. Betsiboka and Mahajamba watersheds, and the unique genotypes they contain, lie in the 9th centre of endemism depicted by^21^. Other unique genotypes from Sariaka and Ambondrobe lie in the 8th centre of endemism^21^. Historical charcoal records suggest that Madagascar experienced a pronounced period of drought and wildfires approximately 5200–5800 years ago^50,51^. Msvar analysis also estimated that *E. madagascariensis* passed through a large bottleneck ~ 5600 years ago, reducing the ancestral population size by ~ 62%. The coincidence of a bottleneck, climate extreme, and RDWs could explain the presence of populations that are distinct on either side of the RDW separating the 8th and 9th centres of endemism^21^. It is likely that after this pronounced drought and population decline, the remnant ancestral *E. madagascariensis* population was located a climate refugia at higher, colder elevations of the RDW. Following the watershed hypothesis, *E. madagascariensis* may have dispersed down either side of the watershed peak as lowland climates become more agreeable. Rere populations separated by this peak would undergo evolution in isolation from one another and undergo genetic drift, becoming the genetically unique populations revealed by our study. Local adaptations to the RDW, developed under natural selection, may have conferred differing fitness to individuals in the areas colonised after the RDW, which have varied climates^21^. This geographic variation in fitness and survival rate may also have contributed to the population divergence observed in this study.

When comparing wild and captive populations, microsatellites revealed a heterozygote excess and a higher average number of alleles per locus in the captive bred turtles compared to the averages for each wild population. The captive breeding population at Ampijoroa was founded from nine individuals from three different localities within ANP^7^. The founding populations (Lakes Ravelobe, Matsaborimavo and Antsiloky) have different allele frequencies and are in Hardy–Weinberg equilibrium. When such populations are interbred or analysed as one, as is the case for Ampijoroa, an excess of heterozygotes are observed due to the Wahlund effect^52^. Despite the high level of heterozygosity in the captive population compared with the wild metapopulation, the ANAPL for the captive population was too low to be representative of the wild population. This indicates that the captive population does not have sufficient diversity to maintain a high level of heterozygosity if breeding continues for several generations without new breeding individuals. The captive population was never intended to maintain genetic diversity for the entire range of *E. madagascariensis*, but as originally planned may be suitable to maintain the genetic diversity of ANP as it has a higher ANAPL than all populations within the reserve, except Antsilomba.

A higher proportion of the wild populations’ nuclear (microsatellite) diversity was represented by the captive bred hatchlings when compared with mitochondrial diversity. A single haplotype was present in the captive born individuals, while nine cytochrome b and ten COI haplotypes were found in the wild samples. It is expected that after a bottleneck event (such as the founding of a captive population) that mitochondrial diversity may be lost faster than nuclear diversity due to the mitochondrial DNA having a four-fold smaller effective population size compared to the nuclear DNA^53^. In this study, the number of individuals sampled for mtDNA was also smaller than microsatellites, and this sampling bias can reduce the number of haplotypes detected. In a review of 18 captive bred species, Witzenberger and Hochkirch^17^ estimated that captive populations founded from < 15 individuals will fail to maintain a desired 90% of natural genetic diversity after 100 years. Long-term maintenance of the species over several generations is not part of the ex situ strategy in Ampijoroa. The low overall genetic variety observed in the captive bred hatchlings at Ampijoroa 21 years after its creation is due to the small number of breeding females, the three original founders. The founder effect may have been exacerbated if the founding females were related and held similar or the same haplotypes, if only one female introduced was capable of breeding, or if one female dominated the breeding. However, no data exists for COI from founding populations—and this would be important to verify. Captive bred individuals from Ampijoroa were observed to have loci in linkage disequilibrium, which can be a sign of non-random and consanguineous mating^54^. Structure plots reveal that the principal cluster of captive bred Reres is only the principal cluster for three out of nine clusters held by the founder individuals from Lake Ravelobe, indicating that these individuals could be mating more often than the others, resulting in genetic drift. Given that turtles are purposefully placed together during breeding season at Ampijoroa^7^, an effort to randomise turtle pairing for breeding may reduce the effects of drift implied by linkage disequilibrium. Enabling multiple pairings may also increase microsatellite diversity, as female turtles may store sperm from multiple males, and high proportions of clutches have been observed to have multiple paternities in the side-necked turtle *Podocnemis expansa*^49,55^.

These findings allow certain wild populations to be prioritised for in situ conservation efforts. The identification of management units (MUs) is an important process in conserving intraspecific genetic diversity^56,57^. MUs will show signs of significant reproductive isolation from other conspecific populations—namely divergent nuclear allele frequencies and mtDNA—both observed in this study^56,57^. The separate management of populations in the Betsiboka and Mahajamba watersheds from other populations would be supported from this study’s findings. Furthermore, Lakes Ambondrobe and Sariaka should be prioritised as separate MUs, due to their large genetic divergence. Fortunately, Lakes Sariaka and Ambondrobe already exist within protected areas. Lake Sariaka lies within Baly Bay National Park, while Lake Ambondrobe became the first area in Madagascar to be protected for the sake of a single species (the Rere) in 2015. However, there is a large discrepancy in the number of individuals sampled between locations. Over 40 individuals were sampled from Lakes Sariaka and Ambondrobe, while fewer than five were sampled from all other locations along the Maningoza, Manambaho, Manambolo, and Tsiribihina watersheds. In addition, 324 more samples exist from Betsiboka and Mahajamba watersheds, further east, than from across the other watersheds. Consequently, more samples are needed to confidently assign the separate MUs of Betsiboka/Mahajamba, and other watersheds. An analysis of a higher number of markers (e.g. whole genome data or RAD-seq SNPs) would help to confirm this decision. Applying these methods may have the power to detect local adaptation at a finer scale than observed in this study, which would also aid in more precise designation of MUs^22^.

In relation to ex situ conservation at Ampijoroa, captive bred hatchlings hold higher microsatellite genetic diversity than the rest of the wild metapopulation. Higher neutral genetic diversity can be indicative of a higher effective population size (Ne) and lower levels of inbreeding (reviewed in García-Dorado and Caballero^58^). Ne and associated levels of neutral genetic diversity can be predictors of a population’s adaptive potential^58^. For example, in the case of genetic rescue, increasing Ne also increases evolutionary potential^59^. However, neutral genetic diversity is not always correlated with a population’s fitness, as discussed by^60^. There are other important aspects of diversity to consider, such as that held in functionally important genes (e.g. the MHC), when assessing captive population’s viability. The negative effects of captive breeding are well documented in captive and reintroduced species, and a low mitochondrial diversity was observed within captive bred hatchlings^17,61^. If the same individuals are repeatedly bred, over time the turtles released into ANP will all be of a similar genotype, and this could lead to inbreeding and/or genetic drift in the wild. Additionally, lakes within ANP that contain genotypes not found in captive hatchlings (Ankomakoma for example) may have their genetic distinctness diluted by the hatchlings. Ideally, adult breeders from Lakes Ankomakoma, Antsilomba and Ankorovoka, as well as the three original founding localities, would be temporarily taken into the captive colony to broaden the genetic founder basis to encompass all four genetic clusters, three cytochrome *b* haplotypes, and five COI haplotypes found in ANP. Realistically, this would require a much larger captive facility than is currently present (G. Kuchling, *pers. comm*.). As already assumed at the start of this breeding project^7^, turtles bred at Ampijoroa will not be suitable for release outside of ANP, as they may begin to dilute the genetic uniqueness found outside the park—for example at Lakes Sariaka and Ambondrobe. For ex situ conservation to b e applicable for these genetically distinct populations, separately managed breeding programs would be recommended for both. However, as long as the protected status of these populat ions can ensure that the Lakes hold a constant or growing number of Reres, ex situ management may not be necessary.

To conclude, thi s study provides the most comprehensive genetic analysis of *E. madagasc ariensis* to date. Genetic structure is observed in the wild and relates to a division between the Betsiboka and Mahajamba watershed populations from other sampled populations, as well as Lakes Sariaka and Ambondrobe, from which management units have been putatively identified. This structure is hypothesised to originate from a dispersal out of a retreat dispersion watershed after a climate-induced, historic bottleneck event. The captive bred hatchling population at Ampijoroa has maintained high levels of heterozygosity but, as planned from the beginning, does not encapsulate the full scope of genetic variation from the sampled range in the wild. However, the genetic variation of its founding populations is currently also not fully represented. The breeding project would benefit from a temporary integration of additional wild breeders from ANP to broaden the diversity of the captive bred translocation stock. Increased sampling effort to achieve ≥ 25 samples from each locality would help to more accurately resolve population structure^35^. Further sampling from the entire range of the species would be beneficial to provide a complete g enetic analysis with which to direct and implement con servation for all remnant subpopulations. Northern and southern populations of *E. madagascariensis* might be divergent enough to be considered subspecies^7^ and verifying the existence of a subspecies would be fundamental to successful delegation of management units for conservation.

## Supplementary Information

Supplementary Information.

## Data Availability

Datasets associated with this publication (sample information and microsatellite genotypes) are publicly available in Figshare (DOI: 10.6084/m9.figshare.19657491). Mitochondrial haplotype sequences are available in NCBI Genbank with accession codes OL804189–OL804207.
