# Supplementary material for: A population genetic analysis of the Critically Endangered Madagascar big-headed turtle, Erymnochelys madagascariensis across captive and wild populations
Source: Sci Rep. 2022 May 24;12:8740. doi: 10.1038/s41598-022-12422-y (PMC9130144; doi:10.1038/s41598-022-12422-y)
Supplement: Supplementary file 1 — Supplementary Information. [file 41598_2022_12422_MOESM1_ESM.pdf]

Supplementary Information: A Population Genetic Analysis of the Critically Endangered Madagascar big-headed turtle, *Erymnochelys madagascariensis* across Captive and Wild Populations

Nina F. D. White[1,2], Holly Mennell[1] , Georgia Power[1] , Dominic Edwards[1] , Luke Chrimes[1] , Lance Woolaver[3,4] , Juliette Velosoa[3] , Randriamahita[3], Richard Mozavelo[3], Tsilavo Hasina Rafeliarisoa[3,5], Gerald Kuchling[6], Javier Lopez[7], Ernest Bekarany[3], Namotoa Charles[3], Richard Young[3], Richard Lewis[3], Michael W. Bruford[1], Pablo Orozco-terWengel[1]*

* Corresponding author
1 = School of Biosciences, Cardiff University
2 = Institute of Zoology, Zoological Society of London
3 = Durrell Wildlife Conservation Trust
4 = Wildlife Preservation Canada
5 = Biodiversity Conservation Madagascar
6 = School of Biological Sciences, University of Western Australia
7 = Animal Health Department, Chester Zoo

Table S1: Microsatellite Primers. All primers were originally designed by Rafeliarsoa *et al.* (2006). All information in this table is taken from Rafeliarsoa *et al.* (2006), with the exception labels and annealing temperatures (Ta), which were re-optimised by this study. *Complex: $(TC)_8(AC)_7AAAA(TC)_8(AC)_8AATT(TC)_9TT(TC)_8(AC)_{11}$

Primer Name	Forward and Reverse Primer Sequence (5'-3')	Fragment Size (bp)	Repeat Unit	Label	Ta (°C)
**131**	F: AAGTTCAGACTGGGCAGGG<br>R: CCACCTTCAGACACACACTCAC	204–220	$(CA)_{12}$	Fam	
**327**	F: ACACAGGGTCCATCCACTTC<br>R: TCAGCAAAACAAGCAACGAG	308–316	Complex*	Hex	60
**699**	F: CCAGGACATCTTAGACTACTGTTCC<br>R: CACTATTTAGGCTTTTCATTCTGC	225–229	$(GT)_9$	Tam	
**777**	F: GAAAAAAAAAGGGGTGGGG<br>R: AGGGAGTTAGGGGTTGTAGGAG	134–148	$(CA)_{20}$	Hex	
**196**	F: AGGATTCAAACAGTGGAGTGC<br>R: CCCAGACAATGACTAACAAACC	196–220	$(GT)_{21}$	Tam	64
**234**	F: CTCCCACGAAATCTCATGC<br>R: TGTAAGATGCTGGCAAAAGTG	231–235	$(CTTT)_5$	Hex	
**897**	F: TGTGTGGAGAGGGATGGTTC<br>R: GTATGCTTAACCCCCACCTC	147–159	$(GT)_{13}$	Fam	
**499**	F: GTGAGCCCCCAAATSCCC<br>R: TGCTGGACAACTAATCTTTTCTATC	187–205	$(CA)_9GA(CA)_3$<br>$GC(CA)_{14}$	Tam	62

Table S2: Priors and hyperpriors used in Msvar analysis. N_0 = current effective population size, Nt = ancestral population size, t = time of bottleneck, m = mutation rate. M = mean, V = variance. All values are log10.

		**Priors**								**Hyperpriors**							
**Combination**	**Run**	**N_0 M**	**N_0 V**	**N_t M**	**N_t V**	***t* M**	***t* V**	**m M**	**m V**	**N_0 M**	**N_0 V**	**N_t M**	**N_t V**	***t* M**	***t* V**	**m M**	**m V**
1	1	4	1	4	1	5	1	-3.5	1	4	1	4	1	5	1	-3.5	0.25
(2,4,9)	2	3	1	4	1	4	1	-3.5	1	3	1	4	1	4	1	-3.5	0.25
	3	5	1	4	1	4	1	-3.5	1	5	1	4	1	4	1	-3.5	0.25
2	1	5	1	5	1	4	1	-3	1	5	1	5	1	4	1	-3	0.25
(3,5,8)	2	3	1	5	1	4	1	-3	1	3	1	5	1	4	1	-3	0.25
	3	5	1	3	1	3	1	-3	1	5	1	3	1	3	1	-3	0.25
3	1	5	1	4	1	4	1	-3	1	5	1	5	1	4	1	-3	0.25
(3,6,7)	2	3	1	5	1	5	1	-3	1	3	1	5	1	5	1	-3	0.25
	3	5	1	3	1	3	1	-3	1	5	1	3	1	4	1	-3	0.25

Table S3: Mode and 95% highest posterior density intervals for parameters used in MSVar. N_0 = current effective population size, N_t = ancestral population size, mu = mutation rate, t = time of bottleneck. Values are log10.

		**N_0**		**N_t**		**mu**		**t**	
**Population**	**Run**	**Mode**	**HPD**	**Mode**	**HPD**	**Mode**	**HPD**	**Mode**	**HPD**
Ankomakoma	2	2.25	1.32-2.93	4.75	4.05-5.47	-3.68	-4.09 - -3.18	4.25	3.33-4.90
	4	1.65	1.00-2.48	4.65	3.95-5.29	-3.55	-4.01 - -3.09	3.75	3.04-4.42
	9	2.05	1.43-2.85	4.75	4.08-5.44	-3.65	-4.09 - -3.18	4.05	3.40-4.81
Ankorovoka	2	2.35	1.47-3.27	4.75	3.98-5.42	-3.65	-4.07 - -3.15	4.15	3.27-5.07
	4	2.05	1.17-2.97	4.65	3.92-5.35	-3.55	-3.97 - -3.06	3.85	3.01-4.73
	9	2.45	1.41-3.14	4.75	4.07-5.48	-3.55	-4.07 - -3.15	4.35	3.28-4.97
Antsilomba	2	2.35	1.16-2.90	5.05	4.27-5.62	-3.55	-4.07 - -3.17	4.05	3.02-4.70
	4	1.65	0.91-2.58	4.85	4.17-5.60	-3.55	-4.02 - -3.10	3.55	2.78-4.34
	9	1.45	0.73-2.63	5.05	4.38-5.67	-3.63	-4.15 - -3.23	3.35	2.64-4.50
Sariaka	3	1.55	0.77-2.43	4.75	4.07-5.39	-3.28	-3.69 - -2.79	3.75	2.95-4.58
	5	1.35	0.69-2.24	4.65	3.95-5.23	-3.15	-3.60 - -2.68	3.85	2.97-4.45
	8	1.45	0.58-2.13	4.45	3.74-5.09	-3.15	-3.54 - -2.64	3.55	2.75-4.32
Ampijoroa	3	1.75	0.96-2.43	4.65	4.04-5.23	-3.25	-3.72 - -2.81	3.55	2.98-4.36
	5	1.45	0.82-2.18	4.55	3.91-5.19	-3.15	-3.61 - -2.71	3.45	2.84-4.16
	8	1.75	0.74-2.36	4.35	3.75-4.98	-3.05	-3.56 - -2.65	3.55	2.71-4.22
Ambondrobe	3	1.55	0.60-2.35	4.75	4.16-5.38	-3.25	-3.73 - -2.81	3.45	2.61-4.22
	6	1.65	1.07-2.19	4.75	4.14-5.39	-3.15	-3.64 - -2.73	3.65	3.01-4.19
	7	1.55	0.95-2.37	4.55	3.91-5.16	-3.15	-3.59 - -2.69	3.55	2.89-4.28
Andranomiditra	3	1.65	0.99-2.42	4.55	3.90-5.21	-3.25	-3.70 - -2.79	3.85	2.99-4.49
	6	1.65	0.48-2.32	4.45	3.88-5.14	-3.25	-3.68 - -2.76	3.65	2.68-4.39
	7	1.55	0.75-2.30	4.35	3.71-4.92	-3.15	-3.61 - -2.69	3.65	2.83-4.29
**Average**		1.77	0.85-2.33	4.67	3.73-4.95	-3.36	-3.53 - -2.68	3.75	2.74-4.17

Table S4: Analysis of Molecular Variance (AMOVA) results, showing the partition of genetic variation between microsatellite genetic clusters, sampling locations, and individuals. Mitochondrial genetic variation at the cytochrome oxidase I gene (COI) between phylogenetic clade, sampling location, and individuals, and at the cytochrome b (Cyt B) between phylogenetic clade, sampling location, and individuals. D.f. = degrees of freedom.

Microsatellites

Source of variation	d.f.	Sum of squares	Variance components	Percentage of variation
Among groups	5	385	0.43	14
Among populations within groups	17	83	0.19	6
Within populations	861	2200	2.55	81
Total	883	2667	3.17	

COI

Source of variation	d.f.	Sum of squares	Variance components	Percentage of variation
Among groups	1	102.44	10.63	95.27
Among populations within groups	3	3.79	0.039	0.35
Within populations	109	53.38	0.5	4.39
Total	113	159.61	11.16	

CYT B

Source of variation	d.f.	Sum of squares	Variance components	Percentage of variation
Among groups	2	288.5	8.82	92.88
Among populations within groups	12	8.36	0.01	0.1
Within populations	41	27.38	0.67	7.03
Total	55	324.23	9.5	

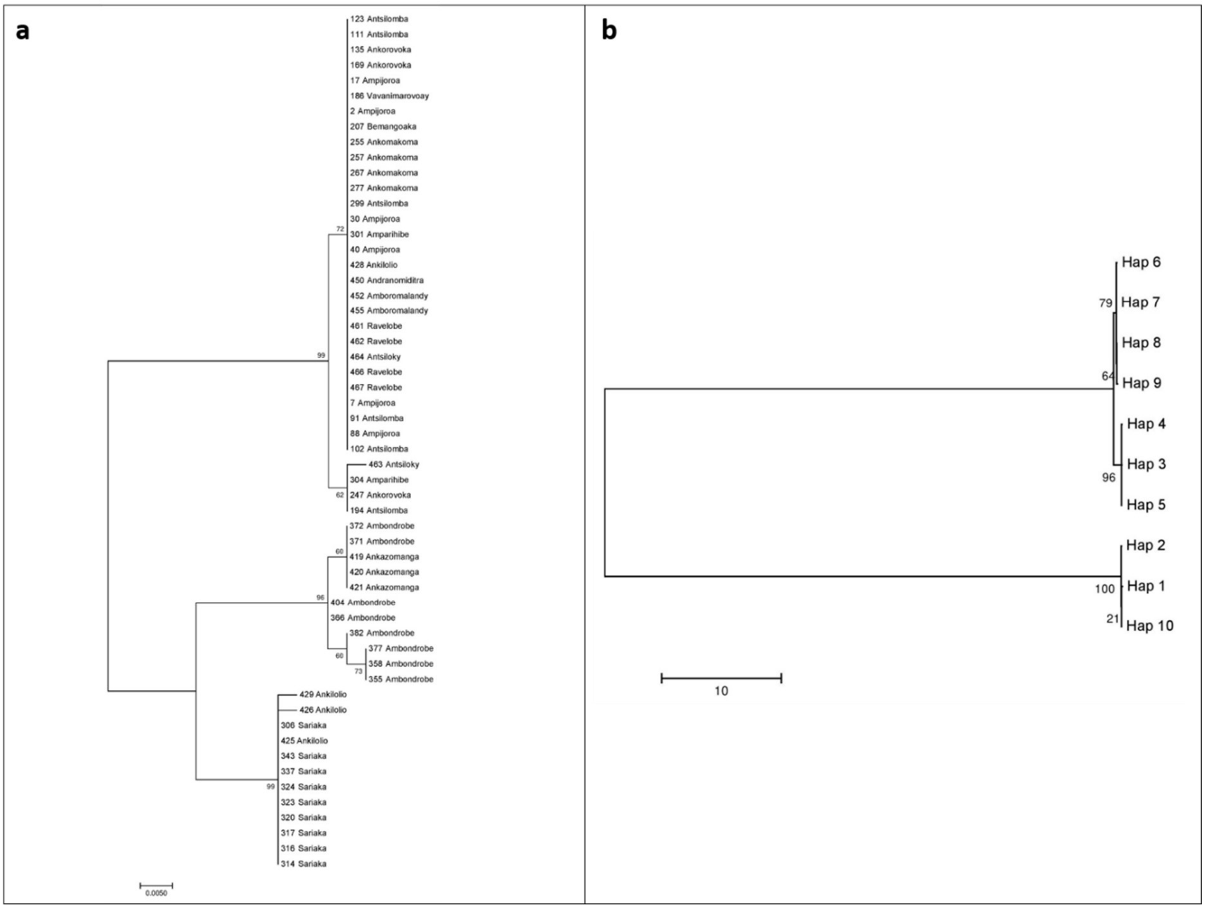

Figure S1: Maximum likelihood trees for cytochrome b data according to a) sampling location and b) haplotype

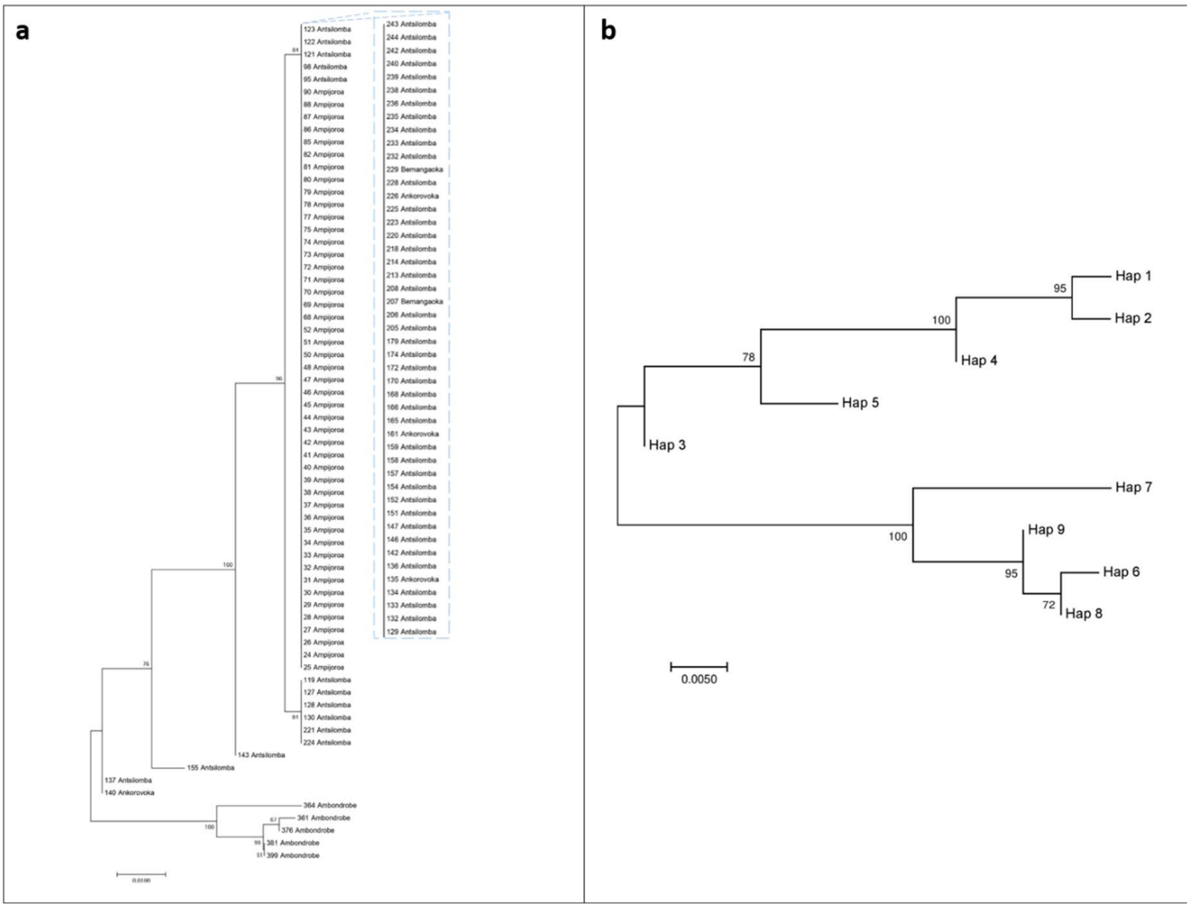

Figure S2: Maximum likelihood trees for cytochrome oxidase I data according to a) sampling location and b) haplotypes. N.B. in panel 'a' the clade containing individuals from Ampijoroa and Antsilomba has had its top half moved to the side of the tree, to enable to figure to be displayed legibly. The moved half is displayed within a blue dashed box, with dashed lines indicating its original position in the tree.
